# Supplementary material for: A structured RNA motif locks Argonaute2:miR-122 onto the 5’ end of the HCV genome
Source: Nat Commun. 2021 Nov 25;12:6836. doi: 10.1038/s41467-021-27177-9 (PMC8616905; doi:10.1038/s41467-021-27177-9)
Supplement: Supplementary file 1 — Supplementary Information [file 41467_2021_27177_MOESM1_ESM.pdf]

Supplementary Figure 1

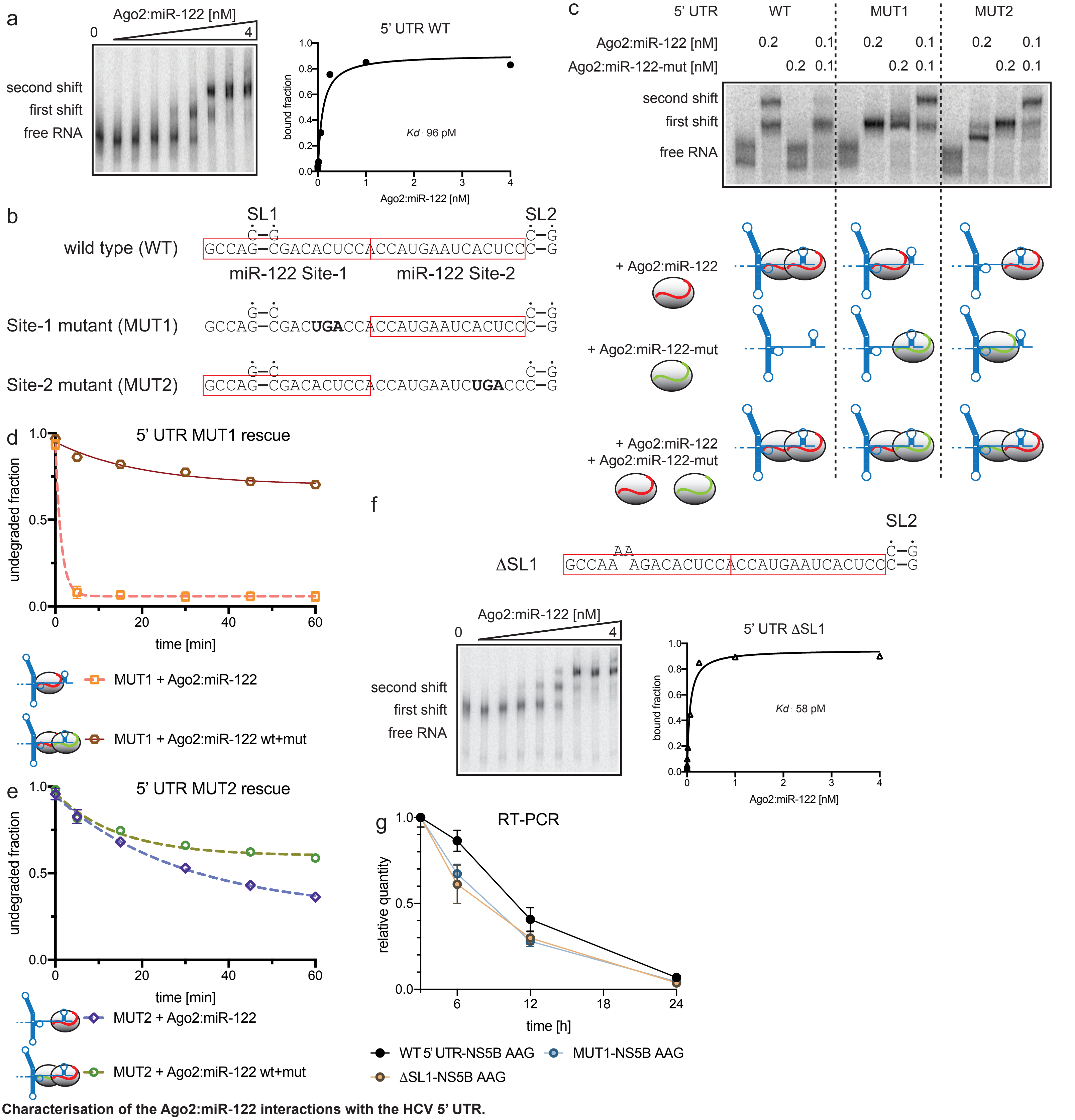

Characterisation of the Ago2:miR-122 interactions with the HCV 5' UTR.
a) Binding of Ago2:miR-122 to the Wild Type HCV 5' UTR in vitro analyzed by EMSA. Fit to a simple binding model yielded an overall Kd of 96(±22) pM (SE). The experiment was repeated twice and a representative gel is shown.
b) WT and mutant sequences (MUT1 and MUT2) used for binding and protection assays. Only two bp of SL1 are shown.
c) EMSA results showing binding to HCV 5' UTR sequences WT, MUT1, and MUT2, by Ago2:miR-122 wt or mut (containing a guide with complementary mutations to the MUT1/MUT2) (top). Diagram depicting proposed binding of HCV 5' UTR constructs by Ago2:miR-122, Ago2:miR-122-mut, or both (bottom). The experiment was repeated twice and a representative gel is shown.
d) Degradation of HCV 5' UTR MUT1 by XRN1 in presence of Ago2:miR-122 wt alone, or Ago2:miR-122 wt plus Ago2:miR-122 mut. Half-life was 1 min for Ago2:miR-122 wt, and 138 min for the wt + mut combination. Data shown as means of 3 independent replicates with error bars representing SEM.
e) Degradation of HCV 5' UTR MUT2 by XRN1 in presence of Ago2:miR-122 wt alone, or Ago2:miR-122 wt plus Ago2:miR-122 mut. Half-life was 41 min for Ago2:miR-122 wt, and 83 min for the wt + mut combination. Data shown as means of 3 independent replicates with error bars representing SEM.
f) Binding of Ago2:miR-122 to the ΔSL1 HCV 5' UTR analyzed by EMSA. The overall Kd is 58(±10) pM (SE). The experiment was repeated twice and a representative gel is shown.
g) Relative abundance over 24 h post-electroporation of replication deficient viral RNA NS5B GDD>AAG with either WT 5' UTR, or the MUT1, or DLS1 mutation. Data shown as means of 3 independent replicates with error bars representing SEM.

Supplementary Figure 2

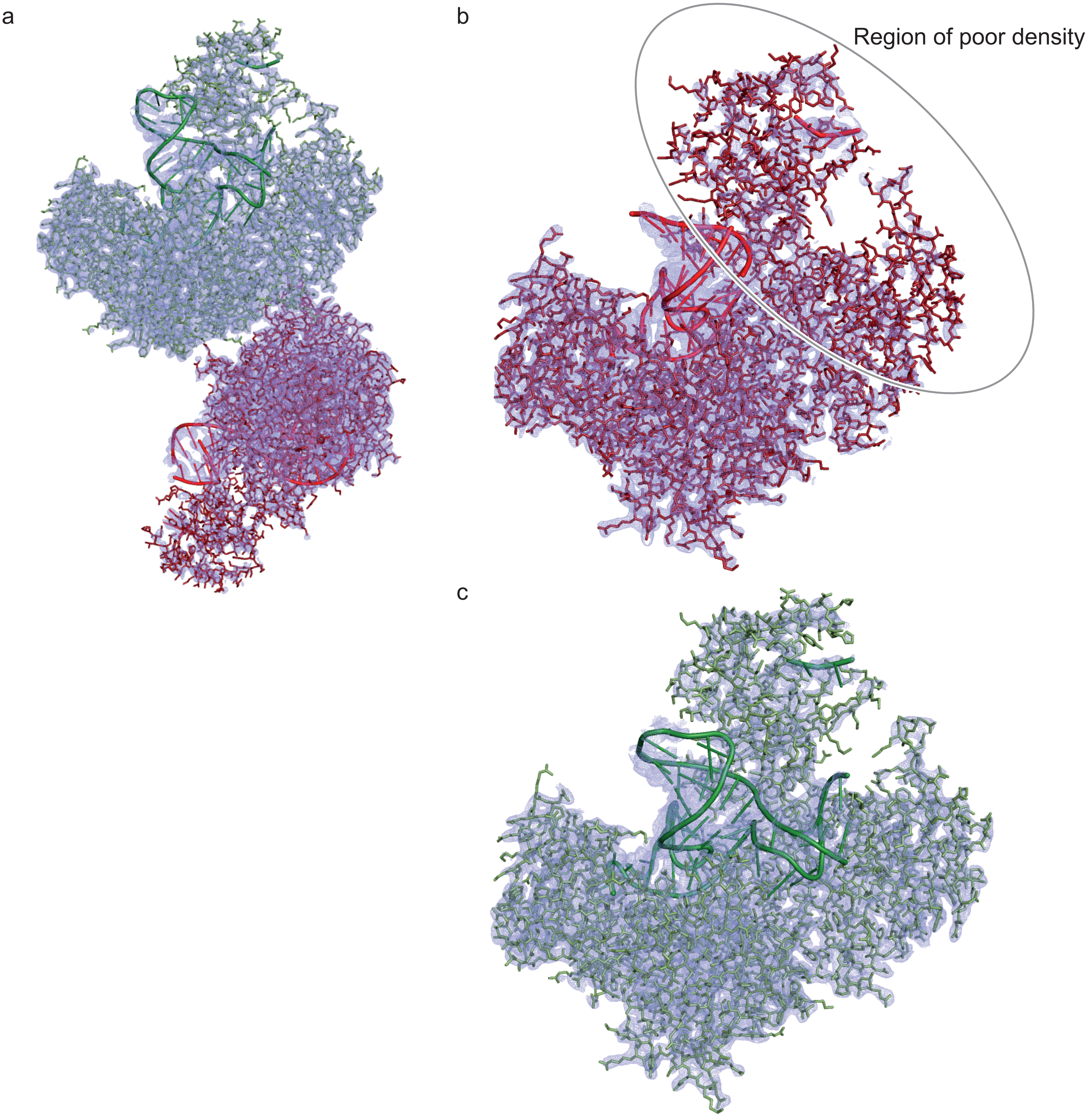

Overview of the asymmetric unit. a) The asymmetric unit comprises two copies of the ternary complex Ago2:miR-122:HCV Site-1. Detail views of **b)** the copy with poor electron density, with the most problematic region circled, and **c)** the copy with good electron density.
2Fo-Fc electron density map contoured at 1 sigma.

Supplementary Figure 3

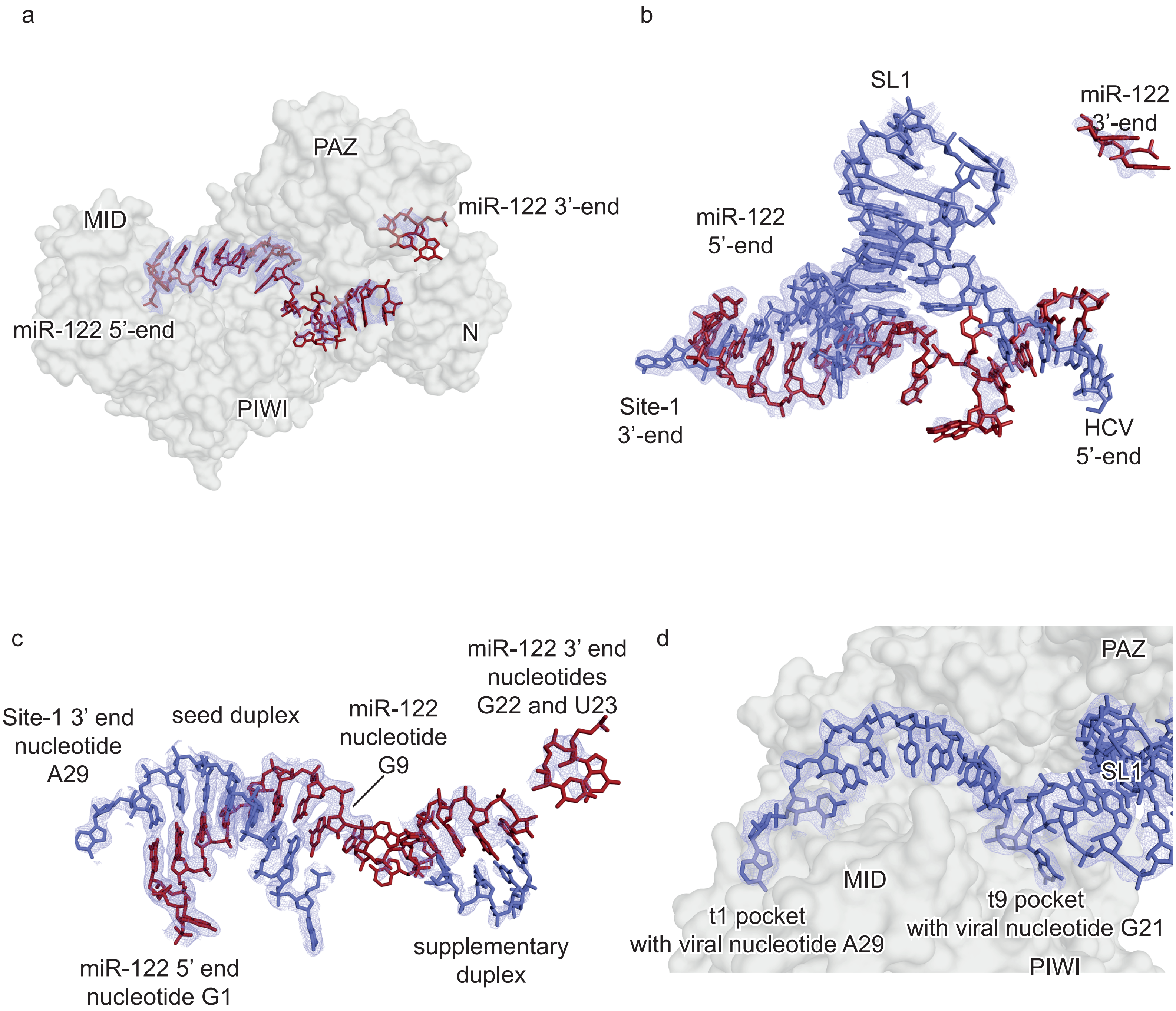

Position within the ternary complex and electron density of miR-122 and HCV Site-1.
a) Overview with HCV Site-1 removed: miR-122 threads along the central cleft of Ago2, between the MID-PIWI and the N-PAZ lobes. The electron density shows the miRNA 5' end and the miRNA 3' end bound to their characteristic pockets in the MID domain, and PAZ domain, respectively. **b)** Detail view of the miR-122:HCV Site-1 duplex, with electron density for SL1 visible. **c)** Detail view of the miR-122:HCV Site-1 duplex with SL1 removed. Electron density is visible for the seed region and the supplementary region. miR-122 nucleotide G9 is stacked against the end of the seed duplex. **d)** Detail view of the 3' end of HCV Site-1 bound to Ago2 with miR-122 removed. Viral nucleotide t1/A29 resides in its characteristic pocket, while viral nucleotide t9/G21 is found bound to a previously unknown pocket in the PIWI domain. All maps are 2Fo-Fc at 1 sigma.

Supplementary Figure 4

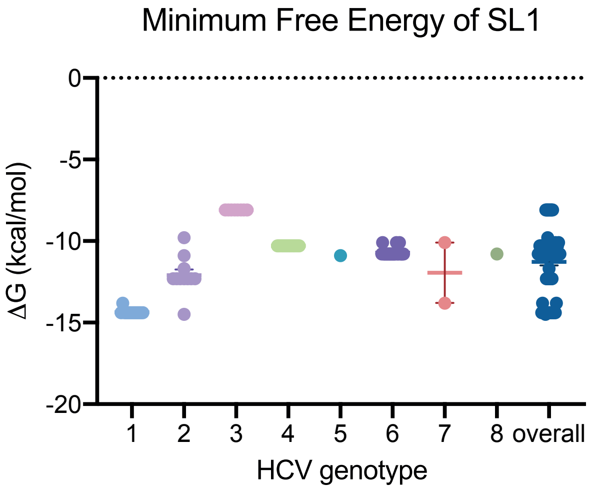

The structural element at Site-1 is conserved across viral genotypes. Minimum free energy of folding of SL1 for all genotype sequences calculated with the ViennaRNA package(Lorenz et al., 2011). Individual data points are sub-genotypes, with means and SE.

Supplementary Figure 5

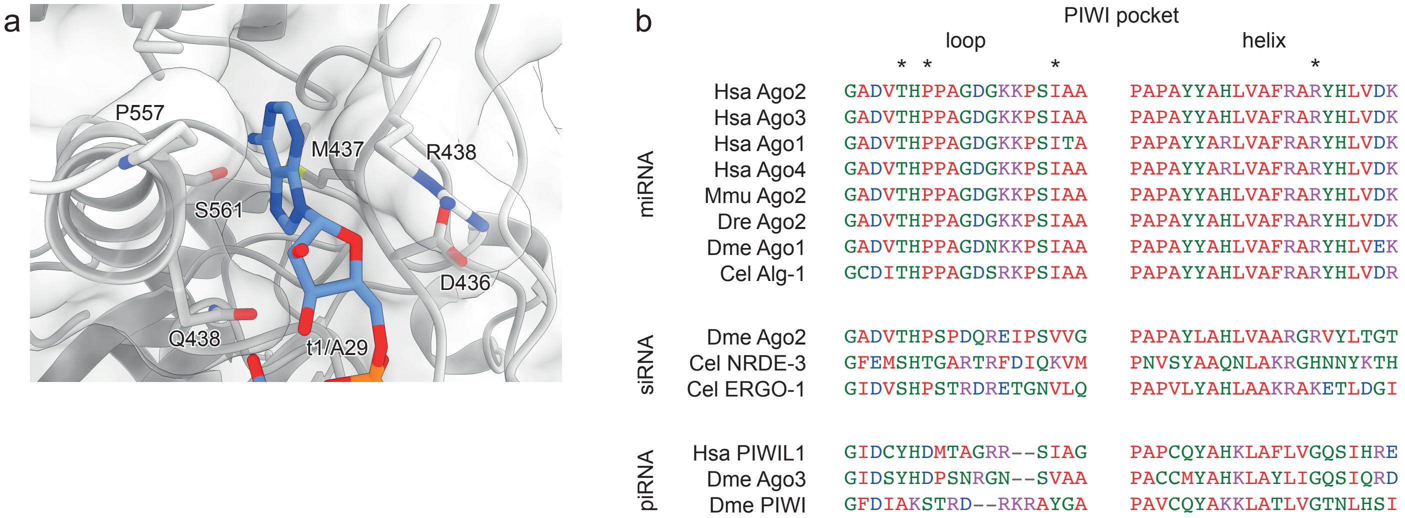

Detail of the t1 pocket in the MID domain. a) Cartoon representation of Ago2 with the residues forming the t1A-binding pocket shown. Similar to the t9-binding pocket, the nucleobase binds between a proline and an arginine residue. **b)** Multiple sequence alignment of the region surrounding the PIWI t9-binding pocket (loop and helix) in human, mouse, zebra fish, fruitfly, and *Caenorhabditis elegans* Ago proteins using primarily miRNA guides, in fruitfly and *C. elegans* Ago proteins using siRNA guides, and in human and fruitfly PIWI proteins using piRNA guides.

Supplementary Figure 6

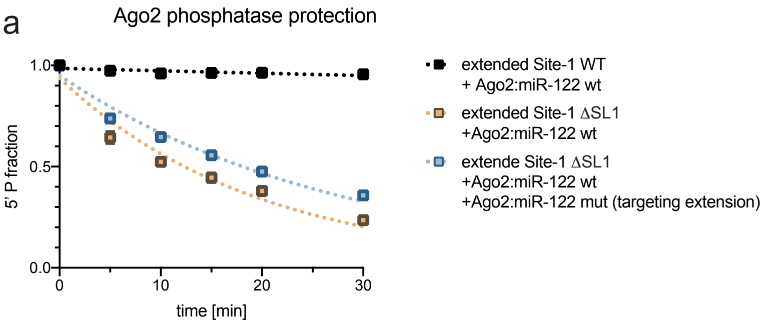

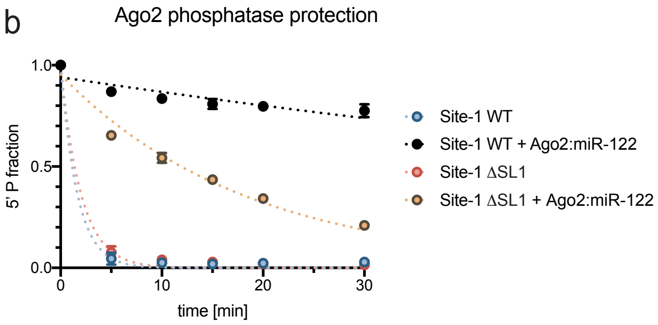

SL1 is necessary for Ago2:miR-122 to protect the HCV RNA 5' phosphate from phosphatase.
a) Enzymatic removal of 5' phosphate from extended Site-1 WT and extended Site-1 ΔSL1 RNAs. Presence of Ago2:miR-122-wt alone, or Ago2:miR122-wt plus Ago2:miR-122-mut (targeting extension) indicated. Half-life was 565 min for extended Site-1 WT, 14 min for extended Site-1 ΔSL1 with Ago2:miR-122-wt, and 20 min for extended Site-1 ΔSL1 with Ago2:miR-122-wt plus Ago2:miR-122-mut.
b) Enzymatic removal of 5' phosphate from Site-1 RNAs in presence or absence of Ago2:miR-122. Half-life was ~1.1 min for Site-1 WT alone, 86 min for Site-1 WT with Ago2:miR-122, ~1.4 min for Site-1 ΔSL1 alone, and 13 min for Site-1 ΔSL1 with Ago2:miR-122
All data points are triplicates with SE as error bars.

Supplementary Figure 7

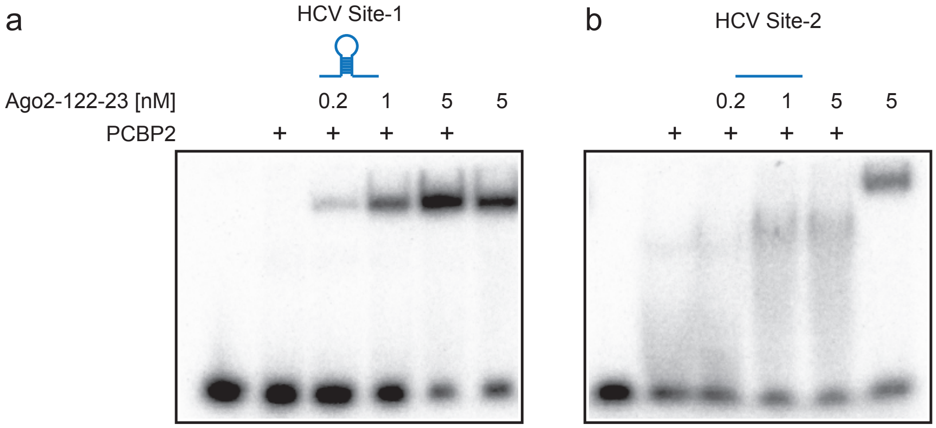

Interaction of HCV Site-1 and HCV Site-2 with PCBP2. a) Analysis of binding of PCBP2 (100 nM) to Site-1 alone and combination with increased concentrations of Ago2:miR-122 analyze by native polyacrylamide gel elecrophoresis. No evidence for binding of PCPB2 to Site-1 is visible, while Ago2:miR-122 leads to a concentration-dependent appearance of a new shift. The experiment was repeated twice and a representative gel is shown. **b)** The same experiment repeated with Site-2. PCBP2 leads to a smeary shift, and appears to compete with Ago2:miR-122. The experiment was repeated twice and a representative gel is shown.

Supplementary Figure 8

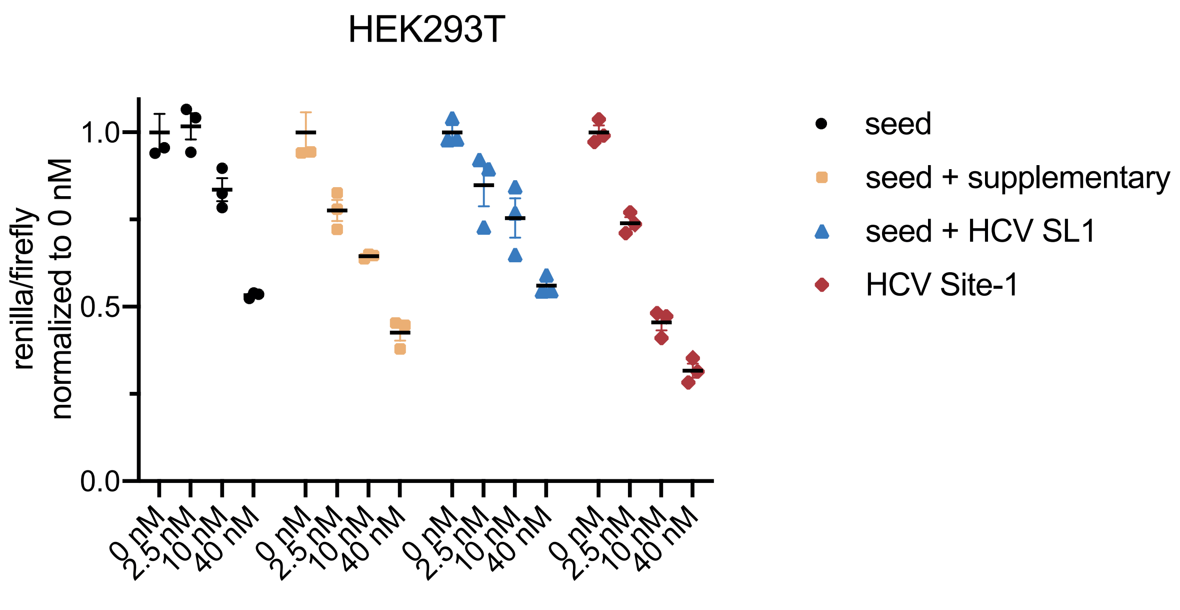

Effect of SL1 on miRNA-mediated silencing in a luciferase reporter system. HEK293T cells were transfected with dual luciferase reporter plasmids containing a single miR-122 seed target in their 3' UTR (seed), a seed target with the HCV supplementary but no SL1, a seed target with SL1 but no supplementary, or the full Site-1. Cells were subsequently transfected with miR-122 duplex to the indicated final concentration. Luciferase activity was assayed and renilla luciferase counts were normalized to firefly luciferase counts and to the 0 nM doses. Individual data points (n=3) with mean and SEM shown.

Supplementary Table 1

Oligonucleotides used in this study

miR-122 guide RNA 23mer
5'-P rUrGrGrArGrUrGrUrGrArCrArArUrGrGrUrGrUrUrUrGrU

miR-122 guide RNA 22mer
5'-P rUrGrGrArGrUrGrUrGrArCrArArUrGrGrUrGrUrUrUrG

miR-122 guide RNA 21mer
5'-P rUrGrGrArGrUrGrUrGrArCrArArUrGrGrUrGrUrUrU

miR-122 capture DNA
5'-Biotin TCTCGTCTAACCATGCCAACACTCCAACTCT

miR-122 competitor DNA
5'Biotin-AGAGTTGGAGTGTTGGCATGGTTAGACGAGA

miR-122 mutant 4-6 guide RNA 23mer
5'-P rUrGrG**rUrCrA**rGrUrGrArCrArArUrGrGrUrGrUrUrUrGrU

miR-122mutant 4-6 capture DNA
5'-Biotin TCTCGTCTAACCATGCCAACTGACCAACTCT

miR-122 mutant 4-6 competitor DNA
5'Biotin-AGAGTTGGTCAGTTGGCATGGTTAGACGAGA

HCV site 1 GC switch
rGrCrCrArGrCrCrGrCrCrUrGrArUrGrGrCrGrGrCrGrArCrArCrUrCrCrA

HCV site 1 GC switch mutated supplementary
rGrArArArGrCrCrGrCrCrUrGrArUrGrGrCrGrGrCrGrArCrArCrUrCrCrA

HCV site 1 deltaSL1
rGrCrCrArArArArArGrArCrArCrUrCrCrA

HCV site 1 GC switch t9 abasic
rGrCrCrArGrCrCrGrCrCrUrGrArUrGrGrCrGrGrCXrArCrArCrUrCrCrA

HCV GC switch site 1 + MUT2
rGrCrCrArGrCrCrGrCrCrUrGrArUrGrGrCrGrGrCrGrArCrArCrUrCrCrArCrCrArUrGrArArUrCrUrGrArCrCrCrC

HCV GC switch site 1 mutated supplementary + MUT2
rGrArArArGrCrCrGrCrCrUrGrArUrGrGrCrGrGrCrGrArCrArCrUrCrCrArCrCrArUrGrArArUrCrUrGrArCrCrCrC

HCV GC switch site 1 deltaSL1 + MUT2
rGrCrCrArArArArArGrArCrArCrUrCrCrArCrCrArUrGrArArUrCrUrGrArCrCrCrC

HCV GC switch site 1 t9 abasic + MUT2
rGrCrCrArGrCrCrGrCrCrUrGrArUrGrGrCrGrGrCXrArCrArCrUrCrCrArCrCrArUrGrArArUrCrUrGrArCrCrCrC

RT PCR primer IRES FWD
CATGGCGTTAGTATGAGTGTCGT

RT PCR primer IRES REV
CCCTATCAGGCAGTACCACAA

RT PCR primer beta actin FWD
GTCACCGGAGTCCATCACG

RT PCR primer beta actin REV
GACCCAGATCATGTTTGAGACC

PCBP2 pET23 FWD cloning primer
ATGGACACCGGTGTGATTGAAG

PCBP2 pET23 REV cloning primer
TTTGCGGCCGCCTAGCTGCTCCCCATGC

T7 FWD primer to prepare templates the in vitro transcription of viral genomic RNA
CCGCTAATACGACTCACTATAGCC

REV primer to prepare templates the in vitro transcription of viral genomic RNA
ACATGATCTGCAGAGAGGCC

HCV 5' UTR MUT1 mutagenesis primer FWD
CCTGATGGGGGCGACTGACCACCATGAATCAC

HCV 5' UTR MUT1 mutagenesis primer REV
GTGATTCATGGTGGTCAGTCGCCCCCATCAGG

HCV 5' UTR MUT2 mutagenesis primer FWD
CTCCACCATGAATCTGACCGGCCGGCATGGTC

HCV 5' UTR MUT2 mutagenesis primer REV
GACCATGCCGGCCGGTCAGATTCATGGTGGAG

HCV 5' UTR ΔSL1 mutagenesis primer FWD
CACTATAGCCAAAAGACTGACCACCATGAATCACTCCG

HCV 5' UTR ΔSL1 mutagenesis primer FWD
CGGAGTGATTCATGGTGGTCAGTCTTTTGGCTATAGTG

NS5B GDD>AAG mutagenesis primer FWD
CGGCTTAGTCGTTATCTGTGAAAGTGC

NS5B GDD>AAG mutagenesis primer REV
GCGGCACACACGAGCATGGTGCA

gBlock for cloning of T7-HCV IRES-HDV into pUC19
AAAAAGCTTTAATACGACTCACTATAGCCAGCCCCCTGATGGGGGCGACACTCCACCATGAATCACTCCCCTGTGA
GGAACTACTGTCTTCACGCAGAAAGCGTCTAGCCATGGCGTTAGTATGAGTGTCGTGCAGCCTCCAGGACCCCCC
CTCCCGGGAGAGCCATAGTGGTCTGCGGAACCGGTGAGTACACCGGAATTGCCAGGACGACCGGGTCCTTTCTT
GGATAAACCCGCTCAATGCCTGGAGATTTGGGCGTGCCCCCGCAAGACTGCTAGCCGAGTAGTGTTGGGTCGCG
AAAGGCCTTGTGGTACTGCCTGATAGGGTGCTTGCGAGTGCCCCGGGAGGTCTCGTAGACCGTGCACCATGAGC
ACGAATCCTAGGCCGGCATGGTCCCAGCCTCCTCGCTGGCGCCGGCTGGGCAACATTCCGAGGGGACCGTCCC
CTCGGTAATGGCGAATGGGACCCAGAATTCAAA
