# Supplementary material for: A structured RNA motif locks Argonaute2:miR-122 onto the 5’ end of the HCV genome
Source: Nat Commun. 2021 Nov 25;12:6836. doi: 10.1038/s41467-021-27177-9 (PMC8616905; doi:10.1038/s41467-021-27177-9)
Supplement: Supplementary file 3 — Reporting Summary [file 41467_2021_27177_MOESM3_ESM.pdf]

# nature portfolio

Corresponding author(s): Ian J. MacRae

Last updated by author(s): Oct 7, 2021



# Reporting Summary

Nature Portfolio wishes to improve the reproducibility of the work that we publish. This form provides structure for consistency and transparency in reporting. For further information on Nature Portfolio policies, see our Editorial Policies and the Editorial Policy Checklist.

## Statistics

For all statistical analyses, confirm that the following items are present in the figure legend, table legend, main text, or Methods section.

| n/a | Confirmed | |
|---|---|---|
| ☐ | ☒ | The exact sample size (*n*) for each experimental group/condition, given as a discrete number and unit of measurement |
| ☐ | ☒ | A statement on whether measurements were taken from distinct samples or whether the same sample was measured repeatedly |
| ☐ | ☒ | The statistical test(s) used AND whether they are one- or two-sided<br>*Only common tests should be described solely by name; describe more complex techniques in the Methods section.* |
| ☒ | ☐ | A description of all covariates tested |
| ☒ | ☐ | A description of any assumptions or corrections, such as tests of normality and adjustment for multiple comparisons |
| ☐ | ☒ | A full description of the statistical parameters including central tendency (e.g. means) or other basic estimates (e.g. regression coefficient) AND variation (e.g. standard deviation) or associated estimates of uncertainty (e.g. confidence intervals) |
| ☒ | ☐ | For null hypothesis testing, the test statistic (e.g. $F$, $t$, $r$) with confidence intervals, effect sizes, degrees of freedom and $P$ value noted<br>*Give P values as exact values whenever suitable.* |
| ☒ | ☐ | For Bayesian analysis, information on the choice of priors and Markov chain Monte Carlo settings |
| ☒ | ☐ | For hierarchical and complex designs, identification of the appropriate level for tests and full reporting of outcomes |
| ☒ | ☐ | Estimates of effect sizes (e.g. Cohen's $d$, Pearson's $r$), indicating how they were calculated |

*Our web collection on statistics for biologists contains articles on many of the points above.*

## Software and code

Policy information about availability of computer code

| | |
|---|---|
| Data collection | The minimum free energy of folding for the stem loops in Supplementary Fig. 4 were generated with the ViennaRNA package 2.0 (Lorenz, et al., 2011). |
| Data analysis | X-ray diffraction data were indexed using DIALS 2.0 (Winter et al., 2018), then scaled and merged with Aimless (Evans and Murshudov, 2013). Initial phases were generated by molecular replacement using Phaser (McCoy, et al., 2007). The model was further built in coot 0.9.1 (Emsley, et al., 2010). Atomic models were assessed using PHENIX 1.14 (Afonine, et al., 2012) and PDB validation servers (www.wwpdb.org). Phosphorscreen intensities were analyzed using ImageQuant version 5.2 (GE Healthcare). All data for plots were analyzed with Prism 9.0 (Graphpad). |

For manuscripts utilizing custom algorithms or software that are central to the research but not yet described in published literature, software must be made available to editors and reviewers. We strongly encourage code deposition in a community repository (e.g. GitHub). See the Nature Portfolio guidelines for submitting code & software for further information.

## Data

Policy information about availability of data

All manuscripts must include a data availability statement. This statement should provide the following information, where applicable:

- Accession codes, unique identifiers, or web links for publicly available datasets
- A description of any restrictions on data availability
- For clinical datasets or third party data, please ensure that the statement adheres to our policy

Atomic coordinates and diffraction data have been deposited in the Protein Data Bank under accession code 7KI3.

# Field-specific reporting

Please select the one below that is the best fit for your research. If you are not sure, read the appropriate sections before making your selection.

- [x] Life sciences
- [ ] Behavioural & social sciences
- [ ] Ecological, evolutionary & environmental sciences

For a reference copy of the document with all sections, see nature.com/documents/nr-reporting-summary-flat.pdf

# Life sciences study design

All studies must disclose on these points even when the disclosure is negative.

| | |
|---|---|
| Sample size | Biochemical experiments were performed in independent triplicates using at least two different preparations of RNA-protein complexes. A sample size of 3 was chosen based on common use in the field. Diffraction data were collected from a single crystal, as only a single crystal of approximately 800 that were screened over the time of the study diffracted to atomic resolution.<br>For diffraction data, 41939 unique reflections were selected based on the resolution cutoff (see Data exclusions). |
| Data exclusions | Diffraction data beyond 3 Å resolution was excluded based on a combination of I/sigma and cc 1/2 values, and through qualitative comparison of electron density maps generated from cutting off resolution at various points. |
| Replication | Biochemical experiments were performed in three independent replicates. Data from cell-based assays were generated from three independent transfections. |
| Randomization | To calculate R-free, out of the 41939 total unique reflections, phenix refine (Afonine, et al., 2012) selected 2058 at random prior to initiation of structural refinement. Otherwise randomization of samples is not relevant to the experiments performed. |
| Blinding | Blinding was not necessary to avoid differential treatment of experimental and control samples. |

# Reporting for specific materials, systems and methods

We require information from authors about some types of materials, experimental systems and methods used in many studies. Here, indicate whether each material, system or method listed is relevant to your study. If you are not sure if a list item applies to your research, read the appropriate section before selecting a response.

## Materials & experimental systems

| n/a | Involved in the study | |
|---|---|---|
| [x] | [ ] | Antibodies |
| [ ] | [x] | Eukaryotic cell lines |
| [x] | [ ] | Palaeontology and archaeology |
| [x] | [ ] | Animals and other organisms |
| [x] | [ ] | Human research participants |
| [x] | [ ] | Clinical data |
| [x] | [ ] | Dual use research of concern |

## Methods

| n/a | Involved in the study | |
|---|---|---|
| [x] | [ ] | ChIP-seq |
| [x] | [ ] | Flow cytometry |
| [x] | [ ] | MRI-based neuroimaging |

## Eukaryotic cell lines

Policy information about cell lines

| | |
|---|---|
| Cell line source(s) | HEK293T cells were purchased from ATCC. Huh-7.5.1 cells were developed at TSRI (Zhong, et al., PNAS, 2005) from the Huh-7.5 cell line provided by Dr. Charles Rice (Rockefeller University). |
| Authentication | No further authentication was performed. |
| Mycoplasma contamination | Cells were routinely tested for mycoplasma every 6 months. |
| Commonly misidentified lines (See ICLAC register) | Not used. |
