# Supplementary material for: A structured RNA motif locks Argonaute2:miR-122 onto the 5’ end of the HCV genome
Source: Nat Commun. 2021 Nov 25;12:6836. doi: 10.1038/s41467-021-27177-9 (PMC8616905; doi:10.1038/s41467-021-27177-9)

Figure 2H uncropped gels

Site-1 no Ago control

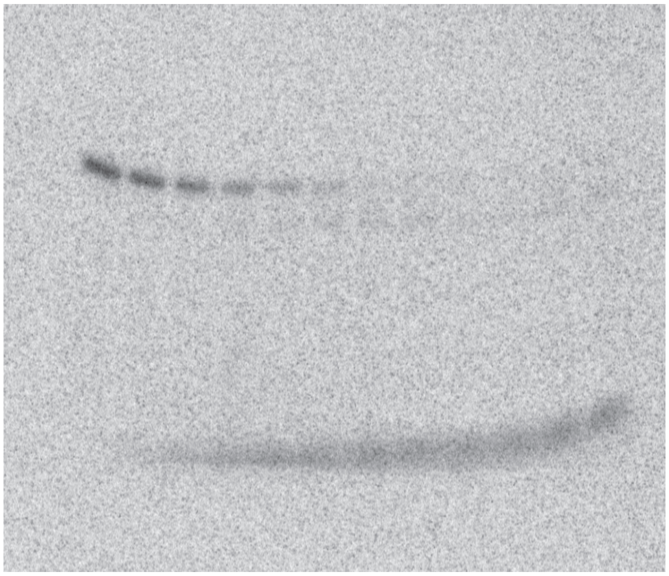

Site-1 + Ago2:miR-122

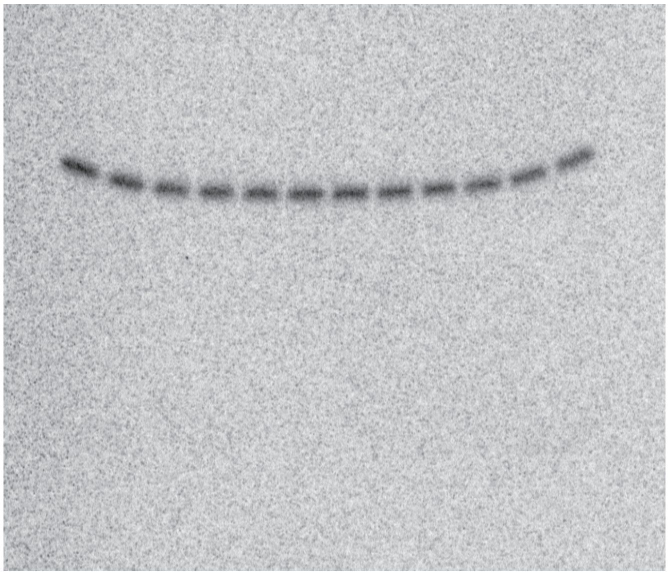

10 U extended Site-1 + Ago2:miR-122

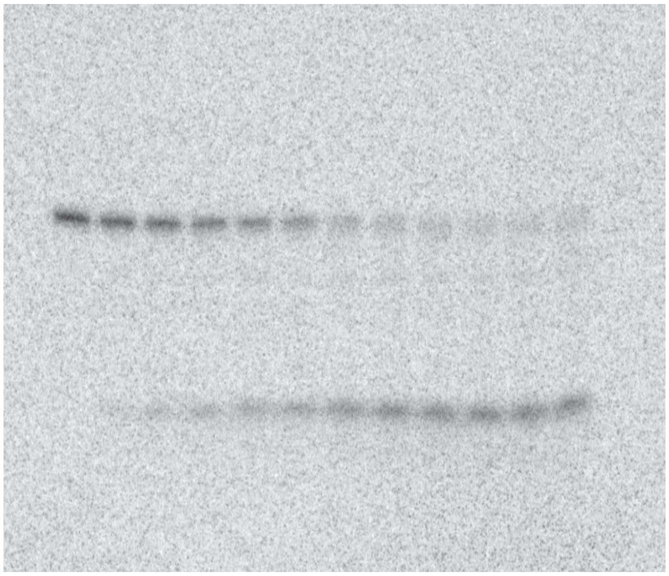

Figure 5A uncropped gels

WT Site-1 and Site-2 construct not used in paper

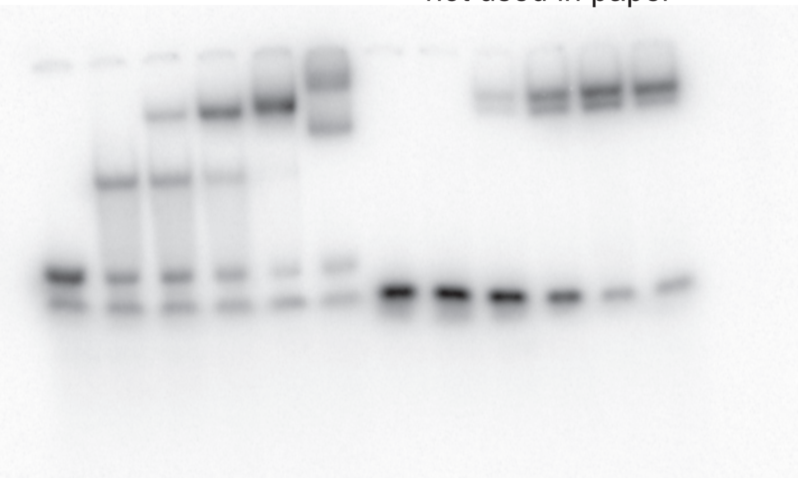

MUT Site-1 and WT Site-2 WT Site-1 and MUT Site-2

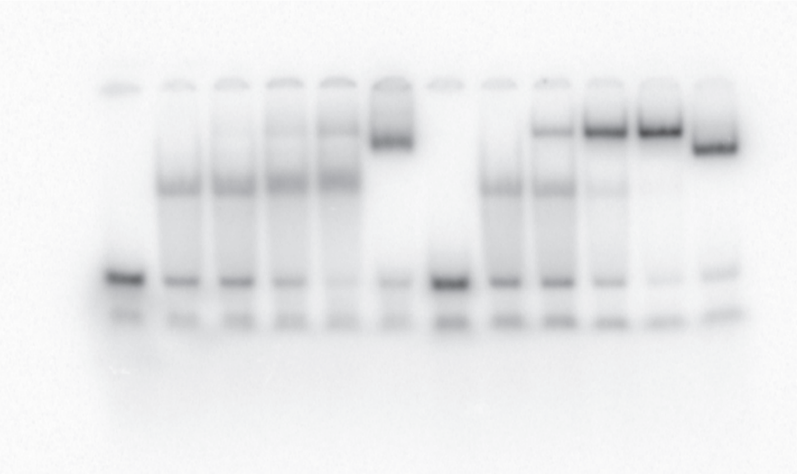

Figure S1C uncropped gel

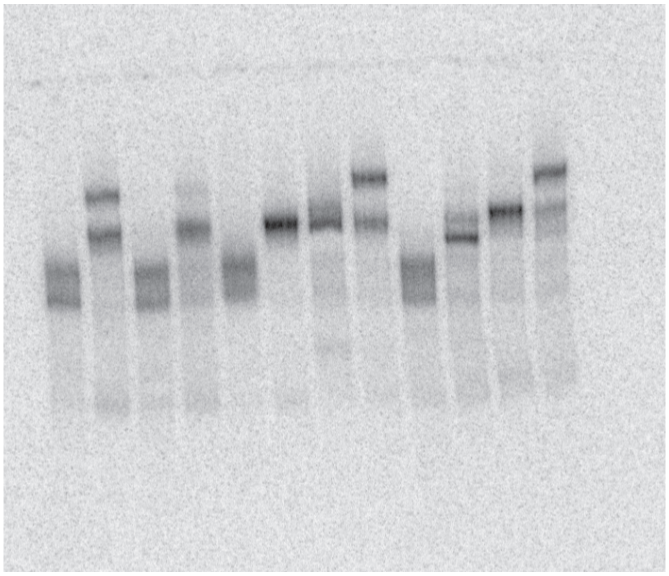

Supplement: Supplementary file 4 — Source Data [file 41467_2021_27177_MOESM4_ESM.zip › Source data uncropped gels.pdf]
